# Integrated Hospital–Territory Organizational Models and the Role of Family and Community Nurses in the Management of Chronic Conditions: A Scoping Review

**DOI:** 10.3390/medicina61071175

**Published:** 2025-06-28

**Authors:** Gianluca Azzellino, Patrizia Vagnarelli, Mauro Passamonti, Luca Mengoli, Lia Ginaldi, Massimo De Martinis

**Affiliations:** 1 Department of Life, Health and Environmental Sciences, University of L’Aquila, 67100 L’Aquila, Italy; lia.ginaldi@univaq.it; 2 U.O.C. Adriatic District Area, AUSL 04 Teramo, 64100 Teramo, Italy; patrizia.vagnarelli@aslteramo.it (P.V.); mauro.passamonti@aslteramo.it (M.P.); 3 Long-Term Care Unit, “Maria SS. dello Splendore” Hospital, AUSL 04 Teramo, 64021 Giulianova, Italy; luca.mengoli@aslteramo.it; 4 Allergy and Clinical Immunology Unit, Center for the Diagnosis and Treatment of Osteoporosis, AUSL 04 Teramo, 64100 Teramo, Italy; 5 UniCamillus-Saint Camillus International University of Health Sciences, 00131 Rome, Italy

**Keywords:** family nurse, community nurse, chronic care, integrated care, hospital discharge, nurse-led, case management

## Abstract

*Background and Objectives*: One of the challenges of modern healthcare systems, in terms of economic and organizational sustainability and the impact on patients’ quality of life, is the progressive increase in chronicity and care complexity. In this scenario, hospital–community integration models represent possible strategies to ensure the continuity of care, reduce readmission rates, and improve clinical outcomes. This study aims to map integrated care models for patients with chronic diseases, with active involvement of the family and community nurse, describing their functions and associated clinical, organizational, and economic outcomes, as well as barriers and facilitators to their implementation. *Materials and Methods*: The review was conducted using the JBI methodology and the PRISMA-ScR protocol and identified 26 studies with a publication range from 2000 to 2025. *Results*: The emerging results highlight the use of integrated and personalized organizational models in the post-discharge phases, with a leading role for the family and community nurse in the assessment, planning, and coordination of various steps. *Conclusions*: The interventions are associated with an increase in patient and caregiver satisfaction, a reduction in outcomes such as the rehospitalization rate, and greater continuity of care.

## 1. Introduction

Aging of the population has led to an increase in the prevalence of multiple diseases, and this represents one of the most significant challenges for modern healthcare systems. Over 50% of adults over 65 live with at least two chronic conditions, a situation that increases the risk of hospital readmissions, loss of autonomy, deterioration in the quality of life, and high healthcare costs [1]. In this new healthcare context, fragmented care models may prove ineffective in ensuring effective, continuous, and patient-centered management [2,3]. Organizational models of hospital–community integration have been developed precisely to respond to this new need. The goal is to make patient care proactive, guiding and facilitating the transition between various care settings, ensuring continuity, coordination, and the personalization of care, especially in such a delicate phase as hospital discharge and returning home [4,5]. In this regard, the literature indicates that these models, especially when they include structured nursing models (e.g., case management and transitional care), can reduce readmission rates, healthcare costs [6], and mortality in elderly patients with chronic diseases [3,7,8,9]. Recent studies emphasize the importance of territorial reorganization strategies and chronic disease management, such as the adoption of telemedicine and the activation of Territorial Operational Centers, with the aim of strengthening the continuity of care and the coordination of integrated care pathways [7]. As highlighted in a recent scoping review, the use of digital and telemedicine tools in transitional care has shown promising results in enhancing the continuity of care, improving patient outcomes, and supporting healthcare professionals in managing postoperative pain at home [8]. Moreover, protected discharge models with the adoption of combined interventions, including nursing follow-ups and caregiver involvement, have proven to be an effective strategy to improve the care experience of frail and chronic patients while also reducing early readmissions [9]. In particular, the role of the family and community nurse plays a key and strategic role in ensuring care transition, acting as a bridge between various hospital and territorial services.

In several healthcare systems (e.g., Italy and Spain), this role is defined as a distinct professional profile that requires specialized post-graduate training in family or community nursing [10]. It differs from generalist community nursing positions by focusing on longitudinal care, proactive case management, and structured support for patients with chronic and complex conditions. However, its scope and implementation vary by country and organizational context [11]. The coordination of care by a responsible and designated healthcare professional is considered one of the crucial elements in the provision of integrated care [12]. Through assessment, planning, education, psychosocial support, and follow-up activities, the nurse figure can contribute to improving therapeutic adherence and self-care by chronic patients [13,14]. However, despite several international experiences and pilot projects, there is still no complete framework that clearly and concisely describes the characteristics of these integrated models that directly involve the community nurse, nor the resulting outcomes. Even the results of studies in the literature are sometimes heterogeneous or limited to specific pathological contexts, such as heart failure or geriatric frailty [5,15]. Moreover, the literature shows a significant lack of standard definitions in care approaches (e.g., nurse-led care, transitional care, integrated care, geriatric team, etc.), making it difficult to compare and use a uniform approach [1,2]. In light of the above, this scoping review aimed to map hospital–community integrated models in the management of chronic diseases with the active involvement of the family nurse, trying to describe the specificity of their role, identify associated clinical, organizational, economic, and experiential outcomes, explore barriers and facilitators to implementation, and assess the level of diffusion, experimentation, or institutionalization in different international healthcare settings.

## 2. Materials and Methods

The study was conducted following the PRISMA-ScR checklist and the methodology proposed by the Joanna Briggs Institute (JBI) for scoping reviews. All of the various methodological steps were carried out with rigidity in order to ensure transparency and reproducibility. The research questions addressed in the review are the following:

- What models of integrated hospital-to-community care are used for the management of chronic conditions with nursing involvement?
- What is the role of the family and community nurse within these organizational models?
- What outcomes (clinical, organizational, economic, or experiential) are associated with such models?
- What barriers and facilitators influence the implementation of these models?

The PCC framework (Population, Concept, Context) was used to define the inclusion criteria:

- Population: Nurses who work in community contexts or in integrated care settings, including family and community nurses.
- Concept: Organizational models for the management of chronic conditions that include an active role of nursing care.
- Context: Any health system, national or local, with no geographical restrictions.

### 2.1. Databases Used and Search Strategy

The bibliographic search was carried out using databases such as PubMed, Scopus, and Web of Science. The search strategy was built using MeSH terms and keywords, combining terms such as “family nurse”, “community nurse”, “chronic care”, “integrated care”, “hospital discharge”, “nurse-led”, and “case management” with Boolean operators.

Articles published from 2000 to the present were included, with the exclusion of letters, editorials, abstracts without full text, and studies focused exclusively on the hospital setting. The complete search strings for each database are available in Supplementary File S1.

### 2.2. Data Extraction

Referring to the JBI methodology for scoping reviews [16], a data extraction table was created for the included studies. A standardized model was used to create the table. The latter included the following elements: author/year, main theme, geographical context, study design and methods, population and sample characteristics, key findings, and research gaps.

### 2.3. Study Selection

Studies were excluded if they did not concern integrated hospital-to-community models or were predominantly hospital-based, if they did not include the nurse as an active part, or if they did not describe outcomes or processes relevant to the role of the family and community nurse.

### 2.4. Screening Process

**Figure 1:**
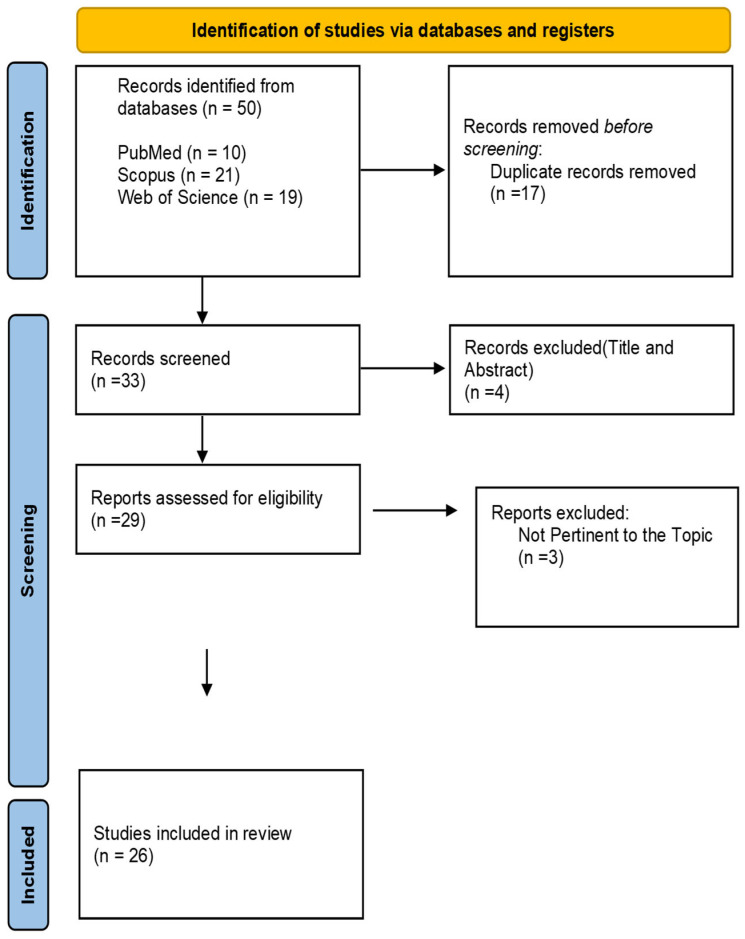
PRISMA 2020 flow diagram illustrating the study selection process. The databases searched were PubMed, Scopus, and Web of Science.

To ensure the accurate inclusion of studies relevant to the review, the process was divided into two phases. In the first phase, two independent reviewers screened the titles and abstracts of the articles identified through the search strategy, based on the selected inclusion and exclusion criteria. In the second phase, the full texts of the studies were read to confirm their compatibility with the objectives of the review. All studies were managed using Zotero (Corporation for Digital Scholarship, George Mason University, Fairfax, VA, USA), available at: https://www.zotero.org/, last accessed 30 April 2025, which supported the authors in organizing citations and full texts throughout the entire scoping review process. Discrepancies between the two reviewers were resolved with the involvement of three additional reviewers. The selection process is illustrated in the PRISMA 2020 flow diagram (Figure 1), in accordance with the PRISMA-ScR protocol [17].

### 2.5. Quality Assessment and Risk of Bias

The risk of bias assessment was not performed for this study, as it is not required for a scoping review. The aim of this study was to map and analyze the available evidence, rather than to critically evaluate each individual study. The authors’ decision is consistent with the methodological guidelines for scoping reviews, as outlined in the *JBI Manual for Evidence Synthesis* and the *PRISMA-ScR*.

### 2.6. Data Synthesis

The results of the included studies were synthesized following the approach outlined in the *JBI Manual for Evidence Synthesis* (2020) for scoping reviews. A descriptive synthesis, without critical appraisal of methodological quality, was used to extract and organize the data as recommended for this type of review. The synthesis was structured into three phases. In the first phase, after data extraction, two independent reviewers identified key themes by analyzing the content of the studies to determine the characteristics of integrated hospital–community care models, the role of the family and community nurse, and the reported clinical, organizational, and experiential outcomes. In the second phase, the data were grouped thematically, highlighting the following main areas: types of organizational models adopted; functions and competencies assigned to the family and community nurse; reported outcomes (e.g., improved continuity of care, reduced readmissions, and patient and caregiver satisfaction); and barriers and facilitators to implementation.

In the third phase, the results were qualitatively described and synthesized. The authors provided a structured overview of the available evidence, highlighting both the potential and limitations of the analyzed organizational models. Although no formal risk of bias assessment was conducted, the presence of several systematic reviews, randomized controlled trials, and mixed-methods studies suggests a moderate overall strength of evidence. Findings were generally consistent for outcomes such as patient satisfaction and reduced readmissions, while evidence on mortality and cost-effectiveness remained more variable. To support and refine the language and style of the manuscript, the authors used artificial intelligence (AI), specifically ChatGPT-4o (OpenAI, San Francisco, CA, USA), available at: https://openai.com/index/hello-gpt-4o/, last accessed 30 April 2025. All methodological steps and scientific decisions were made solely and exclusively by the authors.

## 3. Results

### 3.1. Selection of Studies

The initial search conducted in electronic databases (PubMed, Scopus, and Web of Science) yielded a total of 50 records (PubMed = 10; Scopus = 21; Web of Science = 19). After the removal of 17 duplicates using Zotero, 33 records were screened by title and abstract. This screening led to the exclusion of four studies. Subsequently, 29 full-text articles were assessed for eligibility. Of these, three were excluded for not meeting the review objectives, specifically for not addressing integrated hospital-to-community care models, lacking active nurse involvement, or not reporting relevant outcomes. A total of 26 studies were included in the final analysis (Table 1). The selection process was conducted independently by two reviewers, and any discrepancies were resolved through discussion and consensus, with the support of three additional reviewers.

### 3.2. Main Results

The analysis of the 26 studies showed significant diversity in organizational models, clinical outcomes, and strategies adopted, but at the same time, it also highlighted common patterns attributable to four thematic areas: organizational models, nursing roles and functions, clinical and organizational outcomes, and barriers/facilitators.

- Integrated organizational models

The most widely implemented models are as follows:

- Nurse case management in the post-discharge phase, implemented through tools such as telephone, home, or outpatient follow-up, has shown significant effectiveness in reducing hospital readmissions [5,26,36].
- The transitional care model, led by specialist nurses or community matrons, has been highlighted as effective in ensuring continuity of care after hospital discharge [2,23,34].
- Multidisciplinary team-based care, through the active and coordinated involvement of physicians, social workers, family/community nurses, and psychologists, has proven to be a valuable approach to improving care continuity and patient outcomes [13,14,31].
- Codesigned or patient-centered models, such as those implemented in heart failure care programs [15] or in neurological transitional care pathways [34], have demonstrated the importance of tailoring interventions to individual needs and preferences.

The hospital-to-community transition is commonly implemented through personalized and structured interventions, based on multidimensional and multiprofessional assessment [4,33].

- Role of the Community/Family Nurse

Nurses play a multifunctional and adaptive role in chronic care management. They assess clinical and social needs and identify frail individuals [21,32]. They are responsible for planning care pathways and for the proactive and personalized management of interventions [22,28]. They support self-care, therapeutic education, and motivational coaching, often focused on chronic conditions [13,25]. They also act as clinical leaders within interdisciplinary teams and in coordinating the hospital-to-community transition [14,36].

- Clinical, organizational, and subjective outcomes

Among the most frequently improved outcomes are the reduction in hospital readmissions within 30 to 90 days [5,26,37]. Across the various models applied, improvements were also observed in perceived quality of care, care continuity, and patient and caregiver satisfaction [15,32,34]. Additionally, the studies report an increase in self-care, disease knowledge, and treatment adherence [13,36]. Notably, a positive impact on healthcare costs and a reduction in length of stay were observed in chronic care systems with well-coordinated and effective models [28,33]. However, effectiveness in terms of mortality, functional outcomes (ADL/IADL), or economic indicators remains less consistent and appears to depend on the intensity and continuity of the intervention [4,5].

- Barriers and Facilitators

Among the barriers identified in the studies are the fragmentation of services in chronic disease management and the poor interoperability between different care settings [28,32]. Another barrier is the lack of standardized clinical tools and uniform training on chronic diseases [13,33], combined with nursing workload overload and cultural resistance to integrated approaches [31]. Among the facilitators, nursing leadership stands out, with strong trust in the case manager’s role in chronic care management and interprofessional collaboration [14,36]. Early discharge planning and the timely activation of hospital–community integration services are also highlighted as enablers [5]. Additionally, the use of modern digital tools and telemonitoring technologies, although still not widely adopted, emerges as a promising facilitator [1].

## 4. Discussion

In current international healthcare contexts, one of the most important, complex, and relevant challenges is the management of chronic patients, not only for the economic sustainability of the system, but above all for the social, organizational, and quality-of-life consequences for patients and caregivers. This study acknowledges the effectiveness of hospital–community integration models under the direct guidance of the family and community nurse as a concrete response to the management of chronicity and as a contribution to the improvement of continuity, quality of care, and satisfaction of patients and caregivers. These models rely on proactive patient management, with the aim of anticipating the patient’s needs through the construction of a personalized care pathway, avoiding the fragmentation of episodic care. In this scenario, the nurse emerges as a key connector between services, supporting the stages of assessment, therapeutic education, follow-up, and coordination between different care settings. It should also be noted that fragmented and uncoordinated organizational models can increase the phenomenon of missed nursing care, with negative repercussions on care quality and patient safety [38]. The results of this scoping review are aligned with multiple findings from the international literature. The study by Deschodt et al. [18], for example, analyzed studies that confirm the importance of the nursing role in integrated chronic care management models. The study by Counsell et al. [39] is consistent with the results of our work, showing how a nurse-led multidisciplinary approach can improve quality of life by reducing emergency department visits. Other studies, such as that of Suiker et al. [40], highlight an improvement in the quality of life in chronic patients managed through multifactorial nursing models. This confirms what emerged in our review in terms of perceived continuity, empowerment, and satisfaction. Similarly, the study by Imhof et al. [41] demonstrates a significant reduction in hospitalizations at three months in chronic patients managed at home by nurses, a result that reinforces the idea of an effective community care model. Another point of reflection comes from the study by Stijnen et al. [42], documenting the positive impact of home visits on the social dimension, an aspect often overlooked by policy-makers, yet relevant to the well-being of chronic and frail patients. However, the diversity of results, as demonstrated by the study of Boult et al. [43], shows that the effectiveness of integrated models also depends on contextual factors, such as staff training, care pathway personalization, and institutional support. Other reviews in the literature confirm the aforementioned findings. McParland et al. [32], through a review, summarized various nursing interventions aimed at patients with multimorbidity, with positive outcomes on quality of life, satisfaction, and the perception of care, reaffirming the centrality of the nurse-led model. Among the studies included in the review, the Guided Care model demonstrated how the activity of the nurse case manager improves the patient experience, enhances self-care, and reduces the use of healthcare services. Along the same lines is the study by Naylor et al. [44], showing how nurse-led management in the early stages post-discharge allows a significant reduction in hospital readmission rates and length of stay, thanks to effective hospital–community continuity. Studies such as that of Rich et al. [45] confirm the above, highlighting the importance and effectiveness of structured education and follow-up in reducing hospital readmission rates in patients with chronic conditions such as heart failure. However, not all studies in the literature confirm favorable outcomes: Weinberger et al. [46], for example, observe improvements in satisfaction but not in readmission rates, emphasizing the importance of the organizational context for the effectiveness of interventions. Further positive confirmations come from a study by Morilla-Herrara et al. [47], which demonstrates that structured discharge planning with connection to community services through models such as nurse case management leads to improvements. A review by Latour et al. [33] focuses on nurse case management in the post-hospital discharge phases in complex and chronic patients, showing positive clinical outcomes and high satisfaction in patients and caregivers. These findings in the literature are integrated with those of our scoping review, where not only clinical benefits emerged, but also relational and organizational ones. Comparing our study with the systematic review by Deschodt et al. [18], interesting data emerge: although aggregate outcomes (mortality, ADL, and hospitalization rate) are not always statistically significant, the effects in terms of quality reported by patients, caregivers, and professionals indicate added value in educational, relational, and organizational terms. This is highly relevant for chronic care management, where therapeutic adherence, patient decision-making capacity, and long-term support are key elements just as important as traditional clinical indicators. In summary, the review confirms the importance of the nursing contribution in the management of frail and chronic patients, and this is not limited only to clinical surveillance but also to the promotion of empowerment, guidance in decision-making processes, and long-term continuity of care. Personalizing the intervention, adopting constant follow-up, and coordinating resources are factors that make holistic care feasible, centered not only on the disease, but on the person and their life network. In this sense, integrated models not only provide an organizational response but also represent a cultural proposal for rethinking chronicity, based on proximity, relationship, and autonomy of the assisted person.

### 4.1. Limitations of the Study

This scoping review was based on indexed sources, with the potential risk of excluding unpublished data or policy documents. The descriptive nature of this review does not allow for quantifying the effectiveness of the analyzed interventions. Moreover, the variability in the study designs included, combined with terminological differences, made it difficult to categorize care models uniformly. The use of AI (ChatGPT-4o) supported the linguistic refinement of the manuscript. While the content and methodological decisions were entirely made by the authors, the influence of AI on language style and phrasing should be acknowledged as a minor aspect potentially affecting narrative consistency.

### 4.2. Future Perspectives

Considering the results that emerged, further studies are needed to deepen the understanding of the effectiveness, sustainability, and transferability of hospital-to-community integrated care models in the management of chronic conditions and frail patients. Longitudinal studies and randomized controlled trials could systematically measure the clinical, economic, and experiential outcomes associated with such models. In the future, it is essential to standardize and develop tools for assessing continuity of care, as well as to define shared roles and nursing competencies across different contexts. Moreover, modern technologies such as telemonitoring, remote monitoring, and the use of artificial intelligence represent a promising perspective that requires further exploration in the context of chronic care management, hospital–community integration processes, and support for patient self-care.

## 5. Conclusions

The scoping review confirms the need for integrated hospital-to-community care models to support frail and chronically ill patients. A key finding of the study is that the leading role in this process must be played by the family and community nurse. Despite the heterogeneity of the included studies, there is a general agreement that nursing activity provides added value in ensuring continuity of care, promoting self-care, and increasing therapeutic adherence and satisfaction among patients and caregivers. The need for standardized post-discharge pathways, the adoption of case management models, and collaboration with other healthcare professionals emerge as fundamental elements to provide effective chronic care. The presence of a healthcare professional in close contact with families within communities, equipped with clinical, educational, and relational skills, proves to be crucial. In managing chronic conditions, it is essential to rely on a healthcare professional who can oversee the transitional phases across different settings, prevent fragmentation and delays, and ensure a holistic approach—leading to high standards of quality and safety in care. However, the study also identifies challenges that need to be addressed. Among these are the lack of standardized models, limited coordination and integration between hospitals and community services, and the need to enhance training and supportive organizational tools. Further robust experimental studies are necessary to assess long-term outcomes such as economic sustainability and patient empowerment.

In conclusion, reducing the gap and finding an effective organizational solution for managing chronic conditions requires the implementation of integrated care models and, above all, recognition of the central role of the family and community nurse. This, however, calls for a cultural shift among policy-makers towards a proactive, sustainable, and holistic healthcare system capable of providing concrete responses to the complex needs of frail and chronic patients.

## Figures and Tables

**Table 1:** Characteristics of included studies (n = 26).

Author/ Year	Main Theme	Geographical Context	Study Type	Sample/Population	Key Findings	Research Gaps
Endalamaw et al., 2024 [1]	Mapping care models for chronic multimorbidity, with a focus on their components, impacts, implementation barriers, and facilitators, especially relevant for low- and middle-income countries	Multinational	Scoping review	54 studies addressing care models for adults with chronic multimorbidity; models covered included integrated, collaborative, nurse-led, chronic, and geriatric models, among others	Care models have been implemented to improve patient satisfaction, cost efficiency, health outcomes, and the overall quality of care. The analysis highlighted several key elements, including multidisciplinary teams, personalized care plans, follow-up, the use of information and communication technologies (ICTs), the involvement of patients and caregivers, leadership, and funding structures. Some critical issues emerged, such as limited resources, team coordination, communication, and access to technology. Among the models analyzed, nurse-led and integrated models proved to be effective and adaptable to various care settings.	Few studies conducted in low-income countries; limited evidence on implementation feasibility in resource-constrained contexts; lack of standardized evaluation tools; need for culturally adapted models and further testing of models in diverse healthcare systems.
Sargent et al., 2007 [2]	Understanding patient and carer perspectives on nurse-led case management for long-term conditions in community settings	United Kingdom	Qualitative study	72 patients and 52 carers receiving community matron case management across six Primary Care Trusts (PCTs)	Five key areas were identified in care delivery based on the principles of case management: (1) clinical care, (2) care coordination, (3) health education, (4) advocacy, and (5) psychosocial support. Patients and caregivers assigned equal importance to psychosocial support and clinical care, often describing “community matrons” as the only reliable source of emotional support. Their role, in terms of advocacy and psychological support, went beyond the recommendations outlined in the Department of Health guidelines.	Discrepancy between official policy definitions and real-world practice (“implementation surplus”); need for inclusion of psychosocial support as a formal competency domain; limited prior exploration of dual support role for both patients and carers in UK community case management.
Ruikes et al., 2016 [4]	To evaluate the effectiveness of an integrated primary care model based on multidisciplinary case management for frail older adults compared to usual care	Netherlands	Cluster controlled trial	536 frail elderly aged ≥70 years (287 in the intervention group, 249 in the control group); identified using the EASY-Care Two-Step screening tool	The use of the CareWell program, designed for primary care targeting frail older adults, included structured interventions such as proactive care planning, case management, medication review, and multidisciplinary team meetings. However, it did not yield significant results regarding functional status (Katz-15), quality of life (EQ-5D), mental health, social functioning, hospital admissions, institutionalization, or mortality. Subgroup analyses also did not show any relevant differences.	Twelve-month duration may be insufficient to show effects; high heterogeneity and individual tailoring of interventions; need for person-centered outcome measures and further longitudinal research on frailty trajectories and timing of interventions.
Takeda et al., 2012 [5]	Effectiveness of different models of clinical service organization in reducing mortality and hospital readmissions among patients with chronic heart failure	Multinational	Systematic review of 25 RCTs	5942 adult patients with chronic heart failure recently discharged from the hospital; follow-up of at least 6 months	There are three care models analyzed in patients with heart failure: case management, outpatient care, and multidisciplinary interventions. In particular, case management models that included specialist nurses, home visits, and telephone follow-ups consistently showed an association with reduced hospital readmissions and all-cause mortality. Other multidisciplinary approaches were also identified as effective in reducing readmissions. In contrast, interventions delivered exclusively in outpatient settings produced mixed results. Key and recurring components of the most effective care models included patient education, medication review, and support for self-management.	Uncertainty remains regarding the optimal components of each intervention; high heterogeneity across studies; lack of standardization in outcomes and intervention reporting; limited evidence on cost-effectiveness and long-term sustainability.
Beaudin et al., 2025 [13]	Factors influencing the integration of self-management support by primary care nurses for patients with coexisting chronic diseases and common mental disorders, and strategies for improvement	Canada	Qualitative interpretive descriptive study	23 primary care nurses with experience in follow-up care for patients with both chronic diseases and common mental disorders	The key determinants influencing the spread of nurse-led self-management support include clinical factors (knowledge, skills, workload, clinical tools, and attitudes), professional factors (roles, interdisciplinary collaboration, and team composition), and regulatory and functional factors (organizational culture and operational mechanisms). Potential improvement strategies include training focused on common mental health disorders, the development of appropriate clinical tools, and clinical support and coaching. All of this requires effective collaboration and cultural change.	Need for targeted training programs and standardized tools for SMS integration; lack of implementation frameworks and cultural safety considerations; future research needed on the effectiveness of digital tools and on the perspectives of other stakeholders including patients.
Shirey et al., 2021 [14]	Development and implementation of a nurse-led, interprofessional collaborative practice (IPCP) model focused on transitional care coordination and chronic disease management for underserved populations	United States	Descriptive implementation study	Uninsured and underinsured adults with diabetes (PATH clinic) and heart failure (HRTSA clinic), mainly African American and low-income, with complex chronic and behavioral health needs	The nurse-led care models analyzed and identified in the included studies showed high heterogeneity in terms of design, target populations, and implementation settings. Key components emerging from the most effective models included multidisciplinary collaboration, person-centered care planning, structured post-discharge follow-up, and support for patient self-management. Nurse-coordinated interventions were associated with improved continuity of care, higher patient satisfaction, and increased self-efficacy. Some studies highlighted a reduction in the unplanned use of healthcare services, such as hospital readmissions and emergency department visits, although with variable evidence. The effectiveness of these models was strongly influenced by contextual factors such as nursing autonomy, interprofessional integration, and system-level support.	Further research needed on scalability, sustainability, and generalizability to other populations and systems; limited empirical evidence linking IPCP to Quadruple Aim outcomes; call for rigorous mixed-methods evaluation and cost-effectiveness analysis across settings.
Koontalay et al., 2025 [15]	Codesign and development of a user-centered, nurse-led chronic care model for patients with heart failure in a limited-resource setting	Thailand	A codesign study	19 participants: 1 heart failure patient, 16 clinicians (nurses, cardiologist, pharmacist, and dietitian), and 2 organizational leaders from a tertiary hospital	Nine models aimed at improving continuity of care were identified. Among them, the most effective and suitable for the management of chronic heart failure (CHF) was nurse-led case management, supported by a multidisciplinary team and characterized by strong integration with community-based services.	Need for future testing and evaluation of the prototype’s impact on patient outcomes, cost-effectiveness, and scalability in other low- and middle-income countries; limited patient participation due to illness/readmission; potential barriers to implementation include resource constraints and hierarchical cultural norms.
Deschodt et al., 2020 [18]	Core components and effects of nurse-led integrated care models for home-dwelling older people	Europe, North America, New Zealand	Systematic review and meta-analysis	19 studies, 22,168 older adults (mostly ≥65 years) living at home	Most nurse-led models were oriented toward a person-centered approach, with individualized care plans and support from multidisciplinary teams. Although many studies reported positive effects on quality of life, mortality, and hospital and emergency department access, meta-analyses did not show overall statistically significant results.	The meta-analyses did not show significant effects on outcomes. Almost no model used implementation theories to explain how the model should work. Aspects such as reimbursement, costs, and the use of technologies (e.g., telemedicine) were rarely included or described.
Davis et al., 2024 [19]	Feasibility of a nurse-led transitional care intervention via telehealth for adult patients with multimorbidity discharged from an acute care hospital	Australia	Feasibility study using mixed methods	21 adult patients (mean age 78 years) with multimorbidity (3 to 10 chronic conditions), followed for 6–10 weeks post-discharge	The study analyzed a nurse-led intervention for patients with multimorbidity, focused on continuity of care and remote transitional support (telehealth). Patients reported high satisfaction, improved access to services, and a reduced perception of readmission risk. In fact, only 24% were actually readmitted. Furthermore, healthcare professionals played an active role in managing hospital flow through the presence of a Transition Coordinator. The intervention proved to be feasible, practical, and adaptable to the clinical context.	Need for a randomized controlled trial to assess effectiveness and cost-efficiency; absence of systematic hospital readmission risk assessment for patients with multimorbidity; inconsistencies in discharge handover to primary care.
Jepma et al., 2021 [20]	Experiences of frail older cardiac patients with a nurse-coordinated transitional care intervention (Cardiac Care Bridge)	Netherlands	Qualitative study	16 frail cardiac patients aged ≥70 years (mean age: 82.4) who had participated in the intervention arm of a transitional care program	Study participants expressed satisfaction with the active support received through post-discharge home visits, which ensured continuity of care. However, recovery experiences varied: some reported physical improvements, while others faced difficulties due to comorbidities or frailty. Home visits conducted by the community nurse had a positive impact, although their added value is often not fully recognized. Individuals who regularly rely on formal or informal support networks tended to be skeptical about the interventions provided by professionals involved in the continuity-of-care team.	Difficulty distinguishing between usual care and the intervention; uncertainty about which patient subgroups benefit most; need to tailor interventions to frailty level, self-management abilities, and existing care networks.
Uittenbroek et al., 2018 [21]	Experiences and role adaptation of case managers in delivering person-centered and integrated care to older adults through the Embrace model	Netherlands	Qualitative study	11 case managers	Case managers have shifted from a task-based model to a person-centered one, focusing on building trust-based relationships, empowerment, and patient autonomy. The main themes that emerged included a new relational approach with older patients, the introduction of new professional roles, the enhancement of skills and knowledge, and the perceived benefits resulting from case management activities. Despite the role not receiving the recognition it deserves, case managers found this position rewarding both personally and professionally.	Need for clearer support structures, integration of guidelines, and better role definition; issues with combining traditional roles and case management; the importance of continuous training and organizational backing to support long-term implementation and identity development in new care models.
Armold, 2017 [22]	Effectiveness of a nurse-led community case management (CCM) program in reducing healthcare utilization in chronically ill adult patients	United States	Retrospective observational study	307 patients with at least one chronic disease; 151 accepted CCM services, 156 refused	Patients who took part in chronic care management (CCM) services experienced a 55% reduction in emergency department visits and a 61% decrease in hospital admissions compared to those who did not participate. A reduction of 47% in non-urgent visits was also observed, although it was not statistically significant. The CCM services, offered free of charge, were delivered by nurses with advanced training and a master’s degree through home visits and care coordination activities.	Lack of demographic data to identify which subgroups benefit most; limited tracking of patients who may have used other health systems or moved; further research needed on the timing and specific interventions that yield the greatest impact.
Davis et al., 2020 [23]	Development of a nurse-led, person-centered care coordination model to improve the continuity of care for people with multimorbidity at the primary–secondary healthcare interface	Australia	Qualitative descriptive	44 stakeholders (nurses, physicians, allied health professionals, consumer advocates, Aboriginal representatives, executives, general practitioners, and academics) participated in forums and a validation workshop	A pragmatic and adaptable nurse-led care coordination model was designed. The model is built around a transversal component (intersectoral multidisciplinary collaboration), four domains (coordination, governance, communication, and culture), and six operational areas. It addresses existing care challenges in light of current models, integrates cultural and governance elements, ensures personalized and person-centered care, and enhances continuity of care across different healthcare settings.	The model’s feasibility and impact are yet to be evaluated in practice (to be addressed in Part 2 of the study); future studies should assess its implementation, sustainability, and effect on health outcomes; need for tools to validate stakeholder input and broader application in diverse care settings.
Chow et al., 2008 [24]	Evaluation of the impact of community nursing services (CNSs) on self-reported health and hospital readmission among chronically ill patients after discharge	Hong Kong, China	Secondary analysis of a randomized controlled trial	46 chronically ill patients	Clinical nurse specialists (CNSs) played an important role in improving the perceived health status of patients with cardiac and respiratory conditions. However, no significant reduction in hospital readmissions was observed. The vast majority of identified issues were related to physiological factors and health-related behaviors. The most commonly adopted nursing intervention was surveillance, followed by health education activities and care procedures. For patients with respiratory conditions, greater emphasis was placed on health education.	Home visits led to significant improvements in self-reported health among patients with respiratory and cardiovascular conditions, but no statistically significant effects were found for hospital readmissions. Additionally, age, gender, and financial status were identified as predictors of self-perceived health.
Chow & Wong, 2010 [25]	A nurse-led case management program with motivational telephone follow-up to improve the quality of life of patients undergoing peritoneal dialysis	Hong Kong	Randomized controlled trial with pre- and post-test designs	85 patients with end-stage renal failure (43 in the intervention group, 42 in the control)	The study found that the nurse-led case management model, followed by motivational telephone follow-ups, significantly improved the quality of life in patients undergoing peritoneal dialysis. Improvements were noted in symptoms, the impact of kidney disease, sleep, pain, emotional well-being, social functioning, and patient satisfaction. Additional positive outcomes included perceived support from healthcare staff, sleep quality, and social functioning. However, no significant differences were observed in physical health or in the general perception of overall health status.	Generalizability limited due to recruitment from only two hospitals; control group also had access to hotline services, which may have diluted differences; further studies needed to assess long-term outcomes, cost-effectiveness, and scalability across broader renal populations.
Chow & Wong, 2014 [26]	Effectiveness of a nurse-led case management program using empowerment strategies to improve outcomes in older adults with multiple chronic conditions after hospital discharge	Hong Kong	Randomized controlled trial	281 older adults	Older adults with at least two chronic conditions received case management interventions, specifically home visits and phone calls. They were randomly assigned to two intervention groups and one control group. The results showed that readmission rates in the intervention groups were significantly reduced within 84 days after discharge. Additionally, these patients reported improvements in self-efficacy, perceived health status, and physical quality of life. No significant differences were found in mental health outcomes. Key elements contributing to the success of post-discharge interventions delivered by nurse case managers included personalization, empowerment, and support for self-management.	Unclear long-term impact beyond 12 weeks; further evaluation needed for cost-effectiveness and scalability; results influenced by nurse–patient relationship and intervention fidelity; generalizability may be limited due to local context and inclusion criteria.
Davis et al., 2019 [27]	Design and implementation of a nurse-led model to enhance continuity of care across health sectors for individuals with multimorbidity	Australia	Mixed-methods study protocol	Patients with multimorbidity discharged from a tertiary hospital	The protocol proposes a flexible, person-centered nurse-led care coordination model. It consists of six phases: assessment during hospitalization, identification of an individualized care plan, post-discharge follow-up, communication with general practitioners, coordination of care across different healthcare settings, and evaluation. Particular emphasis is placed on multidisciplinary collaboration and the integration of governance and cultural aspects within the reference model.	As a study protocol, no clinical outcomes have yet been reported. Future work is needed to evaluate the feasibility, effectiveness, and cost-efficiency of the model in real-world practice, especially its impact on patient outcomes and health system integration.
Davis et al., 2021 [28]	Effectiveness of nurse-led services in achieving continuity of care for chronic disease patients across the primary–secondary healthcare interface	Multinational	Quantitative systematic review	14 studies included with a total of 4090 adult patients (aged 29–95) with chronic diseases	Nurse-led services are associated with improved patient outcomes, such as reduced hospitalizations and readmissions, increased patient satisfaction, enhanced quality of life, better self-management, and improved symptom control. All the analyzed models included interventions that ensured continuity of care—relational, informational, and managerial—but only a few measured continuity as a specific outcome using validated tools.	Need for cost-effectiveness studies and validation of continuity measurement tools.
Grimsmo et al., 2018 [29]	Feasibility of implementing disease-specific clinical pathways in primary care settings, with a focus on multimorbidity and care transitions	Norway	Mixed-methods study (qualitative + quantitative)	155 health professionals and managers in two case studies (qualitative); 214,722 adult inhabitants and 6061 home healthcare patients across four municipalities (quantitative)	The structuring of disease-specific clinical pathways is not suitable for managing chronic patients in the context of primary care. An effective approach is person-centered, using flexible and personalized care pathways based on individual needs rather than a single condition. Disease-specific pathways tend to be too complex and rigid to apply in everyday practice, especially in home care settings where time is limited and flexibility is essential.	Insufficient research on the contextual adaptation of clinical guidelines during care transitions from hospital to home.
Harvey et al., 2017 [30]	Design, implementation, and evaluation of a nurse-led, person-centered integrated care model for people living with long-term conditions (LTCs)	New Zealand	Implementation project with mixed-methods evaluation (qualitative, quantitative, and participatory)	Adults with LTCs, particularly those recently discharged from a hospital or newly diagnosed; focus on populations in high-deprivation areas	The model introduced the role of Liaison Nurse Consultants (LNCs) with the aim of coordinating care between primary and secondary services, thereby reducing hospital admissions. Key aspects examined included continuity of care, quality of life, equity, and health literacy. Preliminary analyses showed a reduction in hospital admissions and greater engagement within disadvantaged communities. This model highlighted the importance of cultural competence, intersectoral collaboration, and patient empowerment.	Full outcome data and long-term results pending.
Gonçalves et al., 2022 [31]	Mapping and classification of nurse-led care management models for hospitalized patients with multimorbidity	Multinational	Scoping review	21 included studies	The review identified three categories of nurse-led care models: (1) nurse-led programs (such as discharge planning activities, outpatient clinics, and targeted interventions), (2) case management models (led by clinical nurse specialists, nurse practitioners, etc.), and (3) models with nurse facilitators (e.g., nurse navigators and care coordinators). All these approaches share the goal of delivering patient-centered care, supporting transitions between care settings, and promoting self-management. However, variability was observed in terms of autonomy, roles, and competencies across the different models.	Lack of standardized definitions for nurse-led care models. Need for clarity on the roles and competencies required.
McParland et al., 2022 [32]	Identification and evaluation of nurse-led interventions for people with multimorbidity, and the outcomes these interventions impact	Multinational	Mixed-methods systematic review	20 studies	Most of the interventions reviewed included case management or transitional care models, all led by nurses with advanced competencies. Common features identified included support for self-management, person-centered care planning, and continuity of care. Positive effects were most evident in patient-reported outcomes, such as perceived quality of care, health-related quality of life, and self-efficacy. However, the impact on healthcare service use, costs, and mortality proved to be more variable.	Few studies used standardized definitions or validated tools for continuity and multimorbidity; more robust evaluations needed to assess long-term and cost-related outcomes.
Latour et al., 2007 [33]	Effectiveness of nurse-led case management for complex ambulatory patients in general health care	Multinational	Systematic review	Ambulatory adults (≥18 years) with complex needs (e.g., multimorbidity, psychiatric comorbidity, and social vulnerabilities), not disease-specific	Post-discharge nursing case management shows - Positive effects on patient satisfaction; - No impact on emergency room visits; - Contrasting results on other clinical outcomes.	Greater clarity is needed in the definitions of “complex patient” and in the standardized evaluation of outcomes. High-quality randomized controlled trials (RCTs) are necessary, with sufficiently long follow-up periods and precise indicators of patient complexity.
McGovern et al., 2018 [34]	Evaluation of a multidisciplinary, nurse-led transitional care program (JUMP) for young adults with chronic neurological conditions moving from pediatric to adult healthcare	France	Descriptive observational study with quantitative outcome assessment (satisfaction survey)	111 patients	In relation to the transition processes of patients with chronic neurological conditions, high levels of satisfaction were reported by both patients (89%) and parents (91%). Key factors contributing to these outcomes included a personalized and multidisciplinary approach, along with the role of Coordination Nurse Specialists (CoNSs), who ensured continuity of care by supporting patients before, during, and after the transition.	Limited generalizability due to single-center design; lack of long-term outcome data; response rate of 48% may bias results; further evaluation needed on adherence, quality of life, and engagement post-transition.
O’Connell et al., 2023 [35]	Assessment of the effects of nurse-assisted and multidisciplinary outpatient follow-up interventions for patients with decompensated liver cirrhosis	Multinational (16 studies from Europe, Asia, the USA, and Australia)	Systematic review	1224 adult patients; 16 included studies	Three main types of interventions were identified: (1) Educational interventions; (2) Nurse-led case management; (3) Standardized hospital follow-up. All these approaches demonstrated at least one improvement in various outcomes, such as reduced mortality, decreased readmissions, increased knowledge, greater self-efficacy, and improved quality of life. In some cases, a favorable cost-effectiveness ratio was also observed. However, none of these models proved to be superior to the others.	Significant heterogeneity across interventions and outcomes; most studies had moderate-to-low methodological quality; need for well-designed RCTs to evaluate real-world effectiveness, especially in personalized nurse-led models for liver disease.
Schraeder et al., 2008 [36]	Effectiveness of a collaborative nurse-led case management model in reducing healthcare utilization and costs for chronically ill older adults in primary care	United States	Non-randomized controlled trial	677 community-dwelling adults aged ≥65 years at high risk for mortality, functional decline, or high healthcare use (400 in intervention group, 277 in control)	The integration of nurse case managers into primary care for frail older adults significantly reduced the risk of rehospitalization among hospitalized patients, improved continuity of care, and contributed to a modest reduction in overall healthcare costs.	Generalizability is limited due to regional scope; non-randomized design may introduce bias; future studies should include broader populations, randomized designs, and long-term outcome evaluations to confirm sustainability and effectiveness.

## Data Availability

No new data were created or analyzed in this study.

## Supplementary Materials

The following supporting information can be downloaded at https://www.mdpi.com/article/10.3390/medicina61071175/s1, Table S1: Supplementary File S1.
