# Supplementary material for: Integrated Hospital–Territory Organizational Models and the Role of Family and Community Nurses in the Management of Chronic Conditions: A Scoping Review
Source: Medicina (Kaunas). 2025 Jun 28;61(7):1175. doi: 10.3390/medicina61071175 (PMC12298380; doi:10.3390/medicina61071175)
Supplement: Supplementary file 1 [file medicina-61-01175-s001.zip › medicina-3671954-supplementary.pdf]

Table S1: Supplementary File S1.

Database	Search String	Articles Found
PUBMED	("Nurses"[Mesh] OR "Community Health Nursing"[Mesh] OR "Primary Care Nursing"[Mesh] OR "family nurse"[tiab] OR "community nurse"[tiab] OR "nurse-led"[tiab] OR "district nurse"[tiab]) AND ("Chronic Disease"[Mesh] OR "chronic care"[tiab] OR "multimorbidity"[tiab] OR "frail elderly"[tiab]) AND ("Integrated Health Care Systems"[Mesh] OR "care transition"[tiab] OR "hospital discharge"[tiab] OR "hospital to home"[tiab] OR "continuity of care"[tiab] OR "transitional care"[tiab]) AND ("nursing role"[tiab] OR "care coordination"[tiab] OR "case management"[tiab] OR "nursing model"[tiab])	10
SCOPUS	TITLE-ABS-KEY ("community nurse" OR "family nurse" OR "primary care nurse" OR "district nurse" OR "nurse-led") AND TITLE-ABS-KEY ("chronic disease" OR "chronic care" OR "multimorbidity" OR "frail elderly") AND TITLE-ABS-KEY ("integrated care" OR "hospital discharge" OR "care transition" OR "hospital to home" OR "continuity of care" OR "transitional care") AND TITLE-ABS-KEY ("nursing role" OR "care coordination" OR "case management" OR "nursing model")	21
WEB OF SCIENCE	TS=("community nurse" OR "family nurse" OR "primary care nurse" OR "district nurse" OR "nurse-led") AND TS=("chronic disease" OR "chronic care" OR "multimorbidity" OR "frail elderly") AND TS=("integrated care" OR "hospital discharge" OR "care transition" OR "hospital to home" OR "continuity of care" OR "transitional care") AND TS=("nursing role" OR "care coordination" OR "case management" OR "nursing model")	19
